# Complete Genome Sequence of a Circulating Hepatitis B Virus Genotype C Strain Isolated from a Chronically Infected Patient Identified at an Outdoor Hospital in Bangladesh

**DOI:** 10.1128/genomeA.01601-17

**Published:** 2018-03-01

**Authors:** Modhusudon Shaha, Keshob Chandra Das, M. Saddam Hossain, Munira Jahan, Abu Hashem, Sabita Rezwana Rahman, M. Salimullah

**Affiliations:** a Microbial Biotechnology Division, National Institute of Biotechnology, Dhaka, Bangladesh; b Molecular Biotechnology Division, National Institute of Biotechnology, Dhaka, Bangladesh; c Department of Virology, Bangabandhu Sheikh Mujib Medical University, Dhaka, Bangladesh; d Department of Microbiology, University of Dhaka, Dhaka, Bangladesh

## Abstract

Hepatitis B virus (HBV) causes significant global health problems despite the presence of a potential vaccine. HBV chronic cases are increasing rapidly in developing countries like Bangladesh. Here, we report the complete genome sequence of an HBV genotype C strain isolated from a chronic patient identified at an outdoor hospital section.

## GENOME ANNOUNCEMENT

Hepatitis B virus (HBV), a partially double-stranded covalently closed circular (ccc) DNA virus belonging to the Hepadnaviridae family, is highly contagious and causes severe liver infection in humans (1). Unlike other DNA viruses, it is genetically diverse, and it includes 10 distinct genotypes (A to J) and multiple subtypes worldwide (2). In Bangladesh, 64.7% of the people have been reported to be exposed to HBV infection (anti-hepatitis B core positive) at least once in their lifetime (3). HBV genotype D is most prevalent and genotype C is least prevalent in Bangladesh (4); however, genotype C strains are responsible for more chronic cases and cases of hepatocellular carcinoma globally than the others (5, 6). Here, we report the complete genome sequence of an HBV genotype C strain, NHB17003, which was isolated from the plasma sample of a patient who was suffering from chronic liver disease and visited an outdoor hospital section on 12 November 2017 with a high titer of HBV.

The viral DNA was extracted directly from the patient’s plasma using a QIAamp MinElute virus spin kit (Qiagen, Germany). Six overlapping amplicons spanning the whole viral genome were generated by PCR and visualized using gel electrophoresis with 1.2% agarose. The PCR products were then purified using a Purelink PCR purification kit (ThermoFisher Scientific, USA) and sequenced using a BigDye Terminator version 3.1 cycle sequencing kit (Applied Biosystems, USA). The assembling of the overlapping sequences and phylogenetic analysis were performed using SeqMan version 7 software (7) and MEGA 6 (8) software, respectively. Analysis of the mutations was performed using the Stanford HBVseq database (https://hivdb.stanford.edu/HBV/HBVseq/development/HBVseq.html). The genotyping of the sequenced genome was performed using the NCBI genotyping tool (9).

The assembled genome of isolate NHB17003, which has a length of 3,215 bp, comprises four overlapping open reading frames that encode several major proteins such as the surface protein (HBsAg), core protein, polymerase protein, and X protein. The surface protein is encoded with three different segments of independent properties, such as large, middle, and small surface proteins. Furthermore, the core protein is encoded with two separate segments, including the precore protein. Phylogenetic analysis based on the complete genome revealed that isolate NHB17003 belongs to the subgenotype C2 and is homologous with isolates from Thailand. Additionally, isolate NHB17003 was observed to have an amino acid substitution, I126T, which was previously reported as an HBsAg escape mutation (4). Although the patient was screened as being negative for HBsAg, the viral load test results were high. This study recommends that patients with a liver disorder consider a PCR test for HBV, even if an HBsAg test is negative.

### Accession number(s).

The complete nucleotide sequence of HBV isolate NHB17003 has been deposited in GenBank under the accession number MG725248.
